# Cofilin regulates axon growth and branching of *Drosophila* γ-neurons

**DOI:** 10.1242/jcs.232595

**Published:** 2020-04-28

**Authors:** Sriram Sudarsanam, Shiri Yaniv, Hagar Meltzer, Oren Schuldiner

**Affiliations:** Department of Molecular Cell Biology, Weizmann Institute of Sciences, Rehovot 7610001, Israel

**Keywords:** Actin dynamics, Axon branching, Cofilin, Twinstar, γ-neurons, Mushroom body, Neuronal remodeling

## Abstract

The mechanisms that control intrinsic axon growth potential, and thus axon regeneration following injury, are not well understood. Developmental axon regrowth of *Drosophila* mushroom body γ-neurons during neuronal remodeling offers a unique opportunity to study the molecular mechanisms controlling intrinsic growth potential. Motivated by the recently uncovered developmental expression atlas of γ-neurons, we here focus on the role of the actin-severing protein cofilin during axon regrowth. We show that Twinstar (Tsr), the fly cofilin, is a crucial regulator of both axon growth and branching during developmental remodeling of γ-neurons. *tsr* mutant axons demonstrate growth defects both *in vivo* and *in vitro*, and also exhibit actin-rich filopodial-like structures at failed branch points *in vivo*. Our data is inconsistent with Tsr being important for increasing G-actin availability. Furthermore, analysis of microtubule localization suggests that Tsr is required for microtubule infiltration into the axon tips and branch points. Taken together, we show that Tsr promotes axon growth and branching, likely by clearing F-actin to facilitate protrusion of microtubules.

## Abstract (precis)

**Highlighted Article:** The actin severing protein Twinstar, the *Drosophila* cofilin, is required for axon growth and branching presumably by clearing actin aggregates to allow microtubule protrusion.

## INTRODUCTION

The limited regeneration of injured adult neurons within central nervous systems is due to a combination of inhibitory environments (Tedeschi and Bradke, 2017) and reduced intrinsic growth ability (Mahar and Cavalli, 2018). Intrinsic growth abilities decrease with age, concomitant with lower regeneration capacity (Wang et al., 2007). Therefore, uncovering the factors that determine intrinsic growth potential in young developing neurons, holds the promise of furthering our understanding regarding factors that normally restrict regeneration.

**Fig. 1.**
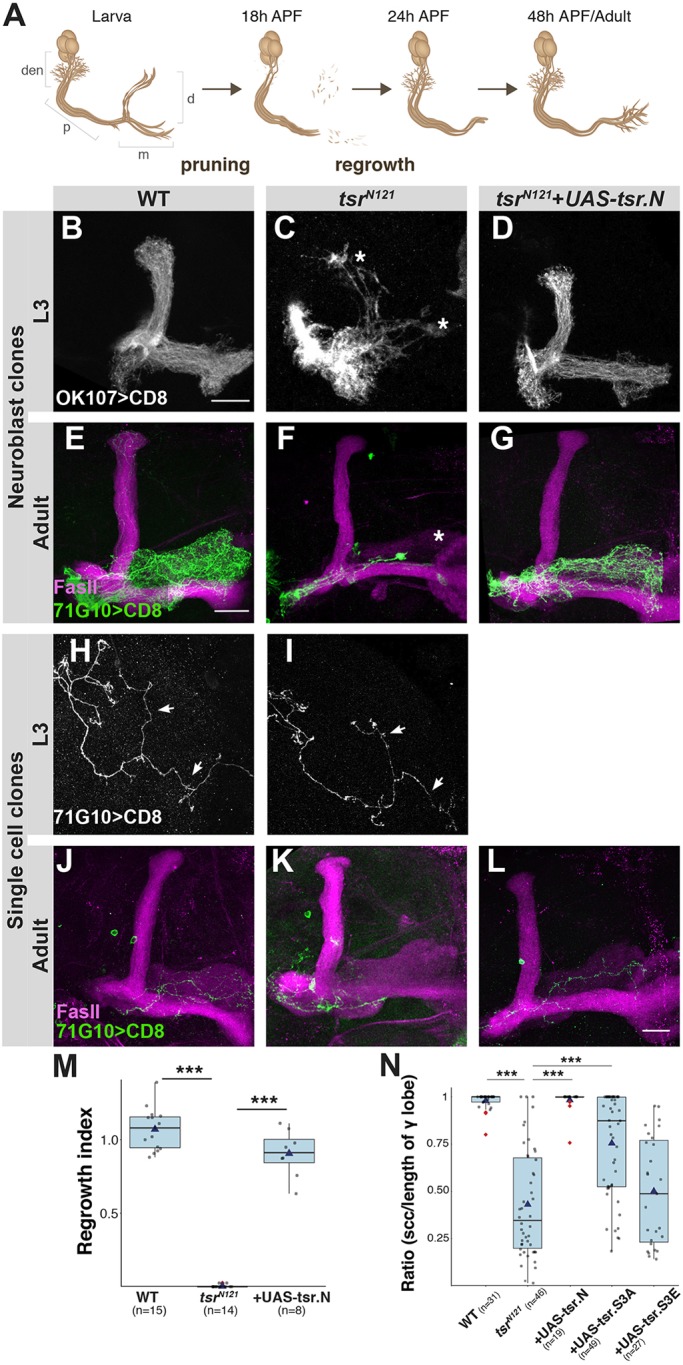
**Tsr is required for axon** **growth of MB γ-neurons.** (A) Schematic representation of γ-neuron developmental remodeling. Den, dendrites; p, peduncle; d, dorsal lobe; m, medial lobe; APF, after puparium formation. (B–L) Confocal *Z*-projections of WT (B,E,H,J), *tsr^N121^* (C,F,I,K) or *tsr^N121^* additionally expressing *UAS-tsr.N* (D,G,L) in γ-neuron MARCM NBCs (B–G) or SCCs (H–I) for L3 or adult flies. Asterisks mark γ-lobe edge. Gray is OK107-Gal4 (B–D)- or R71G10-Gal4 (H–I)-driven mCD8::GFP. Green is R71G10-Gal4 (E–G,J–L)-driven mCD8::GFP. Magenta represents FasII staining. (M) Box-plot quantification of γ-axon regrowth, depicted as a regrowth index. See Yaniv et al. (2012) for quantification method. (N) Box-plot quantification of phenotypic severity shown as ratio of the SCC length to the entire lobe length. Scale bars: 20 µm. ***P<0.005.

Neuronal remodeling is a conserved mechanism, which includes the elimination of axons and synaptic connections, often followed by formation of new connections to sculpt mature neural networks (Luo and O'Leary, 2005; Yaron and Schuldiner, 2016). Remodeling of the *Drosophila* mushroom body (MB), a center for olfactory associative learning, is an excellent model to study developmental axon regrowth (from now on – regrowth) as neurons need to rapidly change their growth state from pruning (degeneration) to regrowth (regeneration; Rabinovich et al., 2016). The MB is comprised of three types of sequentially born neurons – γ, α′/β′ and α/β – of which only γ-neurons undergo developmental remodeling (Fig. 1A; Lee et al., 1999). While we have previously shown that axon regrowth is a genetically controlled program, dependent upon the nuclear receptor transcription factors Unfulfilled (UNF, also known as Hr51; Yaniv et al., 2012) and Ecdysone-induced protein 75B (Eip75B; Rabinovich et al., 2016), the molecular machinery that governs growth in this context is largely unknown. Importantly, we have demonstrated that regrowth is not only molecularly distinct from initial axon outgrowth, but also shares molecular mechanisms with regeneration following injury (Yaniv et al., 2012). To continue to dissect the genetic program that controls axon regrowth, we have recently uncovered the detailed transcriptional landscape of developing γ-neurons (Alyagor et al., 2018). We found that several actin regulators exhibit significant expression dynamics during neuronal remodeling, positioning them as candidates for structural components of axon regrowth.

Axon growth is thought to be driven by growth cones that consist of an actin-rich peripheral zone with filopodia and lamellipodia, and a microtubule (MT)-rich central zone. The ability of growth cones to navigate depends on a dynamic interplay between F-actin and MTs (Blanquie and Bradke, 2018). F-actin dynamics is the combined result of nucleation, elongation and severing of filaments (Omotade et al., 2017; Shekhar et al., 2016). The actin depolymerization factor (ADF)/cofilin family consists of small globular proteins (three in mammals) that depolymerize and/or sever F-actin filaments, and are also implicated in G-actin sequestration (Bernstein and Bamburg, 2010; Flynn et al., 2012; Wioland et al., 2017). Counterintuitively, ADF/cofilin proteins are known to enhance axon growth and have been implicated in neurite formation during development (Flynn et al., 2012), as well as growth cone motility (Endo et al., 2003), branching (Bernstein and Bamburg, 2010; Kanellos and Frame, 2016) and, most recently, axon regeneration (Tedeschi et al., 2019). Interestingly, actin stabilization by pharmacological manipulation or by mutating cofilin slows neurite growth and axon regeneration (Flynn et al., 2012; Tedeschi et al., 2019). The two main hypotheses as to how cofilin promotes axon extension are: (1) by increasing F-actin assembly, either by ‘actin treadmilling’ (Shekhar and Carlier, 2017), expected to increase the supply of severed G-actin to the growing barbed ends, or by removal of capping, thus providing new barbed ends available for elongation, and (2) by clearing actin to allow microtubules protrusion (Bradke, 1999).

Twinstar (Tsr, the sole *Drosophila* cofilin), which was shown to bind actin *in vitro* and to promote its depolymerization and severing (Shukla et al., 2018), is highly and dynamically expressed in MB γ-neurons throughout remodeling (Alyagor et al., 2018). Here, we explore its role during axon regrowth and branching.

## RESULTS AND DISCUSSION

### Tsr is required for axon growth of MB γ-neurons

We have recently uncovered the expression profiles of developing MB γ-neurons at a fine temporal resolution (Alyagor et al., 2018). Out of 126 actin-related genes in *Drosophila*, 77 are expressed above a threshold (for more details, see Alyagor et al., 2018), 45 in a dynamic pattern in developing γ-neurons (Fig. S1A; data taken from Alyagor et al., 2018), out of which 21 are expressed in a pattern suggestive of a potential role in axon regrowth. In this study, we decided to focus on the actin filament-severing protein Tsr, the fly cofilin, whose expression is upregulated during metamorphosis (Fig. S1A), with maximal values reached at the onset of regrowth [at 24 h after puparium formation (APF)].

Tsr has been previously shown to be required for axon growth of both γ- and α/β-neurons (Ng and Luo, 2004). Additionally, *tsr* axons displayed abnormal protrusions and swellings. However, the nature of these swellings, and whether Tsr is required for initial axon outgrowth, regrowth or both, remained unknown. We therefore generated *tsr* homozygous mutant clones using the mosaic analysis with a repressible cell marker (MARCM) technique. In line with results from previous studies (Ng and Luo, 2004), MARCM γ-neuron neuroblast clones (NBCs) homozygous for *tsr^N121^* or *tsr^N96A^*, both considered null alleles derived from imprecise excision of the same P-element (but not molecularly defined), displayed reduced cell numbers and a severe growth phenotype in which axons stopped at the branchpoint (Fig. 1E,F,M; Fig. S1B). In order to differentiate between a defect in initial outgrowth or regrowth, we examined clones in third-instar larva (L3) and found that most, but not all, larval *tsr* γ-axons stalled near the peduncular branch point (Fig. 1B,C). The growth defects could be rescued by expressing a full-length Tsr transgene within the mutant cells (Fig. 1D,G,M). To our surprise, NBCs of the later-born α/β neurons appeared normal in *tsr* mutants (Fig. S1C–E), even though Tsr has previously been implicated in their correct growth (Ng and Luo, 2004). This inconsistency could be due to the use of different Gal4 drivers (R44E04-Gal4 in this study versus OK107 in the original paper), and thus labeling different α/β sub-populations, or be due to the timing of the heat-shock induced recombination (L3 here versus pupae in Ng and Luo).

We next examined single-cell clones (SCCs), in which the anatomical resolution is improved. In contrast to what was seen with neuroblast clones, *tsr^N121^* SCCs extended their axons normally at L3 (Fig. 1H,I). The fact that SCCs undergo initial growth normally, in contrast to NBCs, is likely due to protein and/or RNA perdurance of Tsr, a phenomenon that has been previously shown (Yu et al., 2013). Briefly, in NBCs, *tsr* RNA and protein are diluted by numerous cell divisions, while in SCCs gene deletion occurs during the last division and thus protein and RNA might still be present.

The fact that *tsr* SCCs grow normally in larvae (and also at 6 h APF, *n*=17, not shown) but exhibit a varying degree of growth defects in adults (Fig. 1J,K,N; Fig. S1F–J), suggests that Tsr is required for regrowth independently of its requirement during initial axon outgrowth. Furthermore, overexpressing a wild-type (WT) Tsr transgene (*UAS-tsr.N*) within *tsr^N121^* MARCM clones completely rescued the regrowth defect (Fig. 1L,N). However, expression of the phosphomimetic Tsr.S3E, presumed to be inactive (Ng and Luo, 2004), did not (Fig. 1N; Fig. S2B). Interestingly, expression of the non-phosphorylatable Tsr.S3A, presumed to be constitutively active, resulted in partial growth rescue (Fig. 1N; Fig. S2A), suggesting that phosphoregulation of Tsr is critical for its function during axon regrowth.

### Tsr promotes branching of adult γ-neurons

**Fig. 2.**
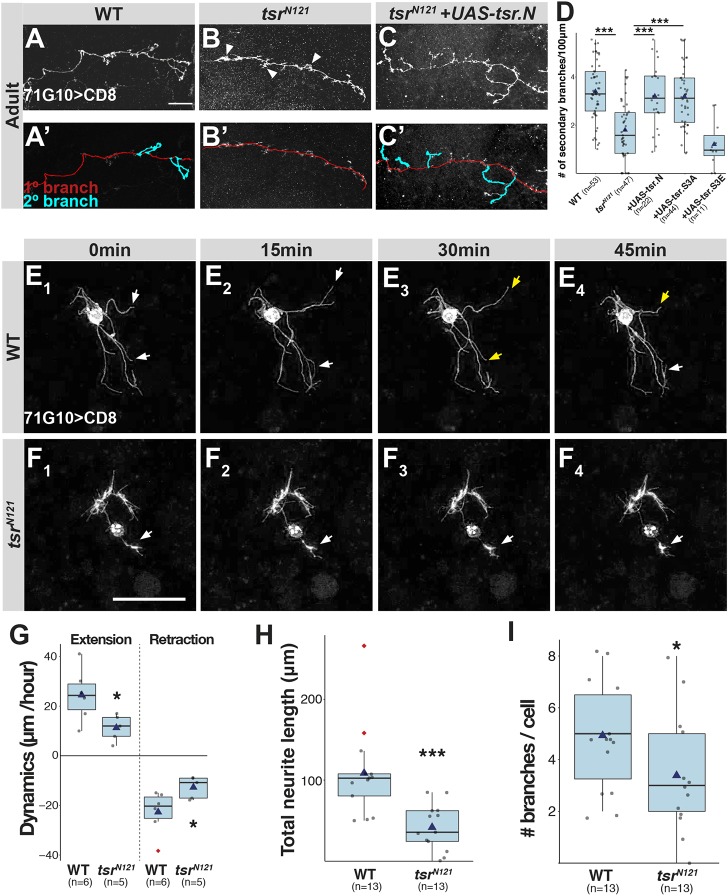
**Tsr is required for γ-neuron branch dynamics.** (A–C) Confocal *Z*-projections of WT (A), *tsr^N121^* (B) or *tsr^N121^* additionally expressing *UAS-tsr.N* (C) γ-neuron MARCM SCCs. A′–C′ are tracings of the primary axon in red, with secondary branches in cyan. Arrowheads point to axonal structures that appear to be failed branch points. Gray is R71G10-Gal4-driven mCD8::GFP. (D) Box-plot quantification of secondary branches (>5 µm) per 100 µm of primary axon. (E,F) Time-lapse series (15 min increments) of confocal Z-projections of WT (E) or *tsr^N121^* (F) primary γ-neurons derived from L3 brains. White and yellow arrows point to extending or retracting branches, respectively. Gray is R71G10-Gal4-driven mCD8::GFP. (G) Box-plot quantification of cell dynamics expressed as change in neurite length divided into extensions and retractions per 1 h imaging averaged over 3 h. (H) Box-plot quantifications of total neurite length per cell. (I) Box-plot quantification of number of branches per cell**.** Scale bars: 20 µm. *P<0.05; ***P<0.005.

While examining *tsr^N121^* SCCs, we noticed that even axons that extended almost normally exhibited elevated occurrences of swellings that resemble lamellipodia-like protrusions. To test whether these might represent failed branching sites, we quantified branches (>5 µm) and found that *tsr* mutant axons had significantly fewer branches than WT axons (Fig. 2A,B,D). Consistent with their effect on axon growth, expression of both *UAS-tsr.N* and *UAS-tsr.S3A* within *tsr* mutant axons rescued the branching defect, interestingly to a similar extent, while *UAS-tsr.S3E* did not (Fig. 2C,D; Fig. S3).

Taken together, we propose that Tsr promotes axon elongation as well as branching. Whether these two functions are linked and utilize the same mechanism of action remains unclear. Interestingly, a recent study suggests that axon branching and growth of γ-neurons could be linked. The authors propose that branching allows axons to bypass obstacles more efficiently (Razetti et al., 2018). In addition, a recent paper demonstrated that the MT regulator Efa6 is also required for both normal branching and growth, further implying that these two processes may be mechanistically related, even in cultured neurons, where presumably the extracellular environment plays a lesser role (Qu et al., 2019). Even in NBCs, *tsr* mutant axons predominantly stop at the branch point, suggesting that branching is key to successful growth. This might also be consistent with the lack of growth defects in α/β neurons as they have much simpler morphology.

### *Tsr* mutant γ-neurons show reduced neurite dynamics

To further probe the effects of Tsr on growth dynamics, we wanted to subject neurons undergoing growth to time-lapse imaging. Unfortunately, live imaging of SCCs during remodeling is practically impossible because the process occurs in a deep pupal structure. Likewise, SCCs are extremely difficult to visualize in the *ex vivo* brain culture system that we have devised (Rabinovich et al., 2015). In order to circumvent this, we turned to primary cultures of sparsely labeled but densely plated WT or mutant γ-neurons (Marmor-Kollet and Schuldiner, 2016). Brains containing WT or *tsr^N121^* MARCM clones were dissociated and plated for 2 days *in vitro* (DIV) before being subjected to time-lapse imaging (Fig. 2E,F). WT neurons exhibited dynamic extension–retraction (Fig. 2E,G; Movie 1), while, in contrast, *tsr^N121^* neurons exhibited significantly reduced neurite dynamics (Fig. 2F,G; Movie 2). Additionally, *tsr^N121^* neurons grew shorter neurites (Fig. 2H) and branched less (Fig. 2I), which is similar to the *in vivo* effects shown above. Additionally, subjection of primary cultures of WT γ-neurons to latrunculin (known to promote actin disassembly, Spector et al., 1983) or jasplakinolide (known to interfere with disassembly, Cramer, 1999) both resulted in decreased sprouting and growth *in vitro* (Fig. S3C–F). Taken together, these data suggest that precise actin dynamics and the balance between assembly and disassembly are crucial for normal neurite extension and branching.

### Tsr does not promote regrowth by increasing G-actin or barbed ends availability

**Fig. 3.**
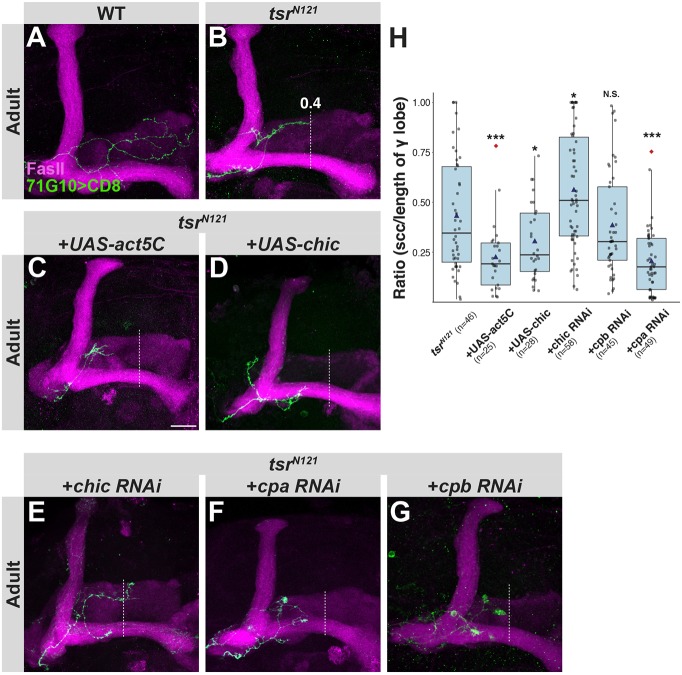
**Tsr does not promote regrowth by increasing G-actin levels or by uncapping.** (A–G) Confocal *Z*-projections of WT (A), *tsr^N121^* (B) or *tsr^N121^* additionally expressing *UAS-act5c* (C), *UAS-Chic* (D), *chic RNAi* (E), *cpa* RNAi (F) or *cpb RNAi* (G) in γ-neuron MARCM SCCs. The dashed line represents the average ratio of *tsr^N121^* SCC to the length of the γ lobe, which is 0.4. Green is R71G10-Gal4-driven mCD8::GFP. Magenta represents FasII staining. (H) Box-plot quantification of phenotypic severity shown as ratio of SCC length to entire lobe length. Scale bar: 20 µm. *P<0.05; ***P<0.005; N.S., not significant.

To inspect the hypothesis that Tsr promotes regrowth by increasing G-actin levels, as suggested by the ‘treadmilling’ model (Shekhar and Carlier, 2017), we performed epistasis experiments in which we aimed to increase the availability of G-actin. Increasing G-actin availability by overexpressing either Act5C (the fly ortholog of β-actin) or Chic, the fly profilin (which delivers G-actin to actin elongation factors), both significantly exacerbated the regrowth defect of *tsr^N121^* SCCs, in contrast to our original prediction based on the aforementioned hypothesis (Fig. 3A–D,H; Fig. S4A,B). Based on these results, we wanted to determine whether profilin and cofilin antagonize each other in the context of axon regrowth. In a parallel project, we demonstrated that perturbing Chic (by RNAi or mutant analysis) also results in a severe γ-axon regrowth defect (Yaniv et al., 2020; Fig. S4C), which can be rescued by overexpressing UAS-Chic. Accordingly, and again in contrast to our original prediction, knocking down Chic levels by RNAi within *tsr* mutant clones ameliorated growth defects (Fig. 3E,H), indeed suggesting they counteract the function of each other.

Next, we aimed to examine the hypothesis that Tsr-mediated severing might promote growth by increasing the fraction of barbed ends that are accessible to polymerization. We thus expressed RNAi transgenes targeting capping protein-α (Cpa) and capping protein-β (Cpb) (Ogienko et al., 2013). Inconsistent with the above hypothesis, we found that knocking down Cpa (but not Cpb) enhanced the *tsr^N121^* regrowth defect (Fig. 3F–H, Fig. S4D). Given that Cpa and Cpb are known to function as an obligatory heterodimer (Schafer and Cooper, 1995), our results probably stem from different RNAi efficiency, something we unfortunately cannot test due to unavailability of antibodies targeting capping proteins.

Taken together, these results suggest that increasing G-actin concentration (by overexpressing Act5C or Chic) or increasing the availability of barbed ends (through knockdown of Cpa) exacerbates growth defects, while reducing F-actin (by knocking down Chic) ameliorates it. Unfortunately, the currently available reporters to examine the G-actin to F-actin ratio did not result in meaningful results due to high background, likely due to the dense neuropil structure. Nonetheless, together these data suggest that Tsr does not promote regrowth by increasing actin polymerization. Instead, since increasing G-actin further impairs regrowth in Tsr mutants, we suggest that Tsr may be important for clearing actin polymers.

### Microtubule localization is disrupted in *tsr* mutants

**Fig. 4.**
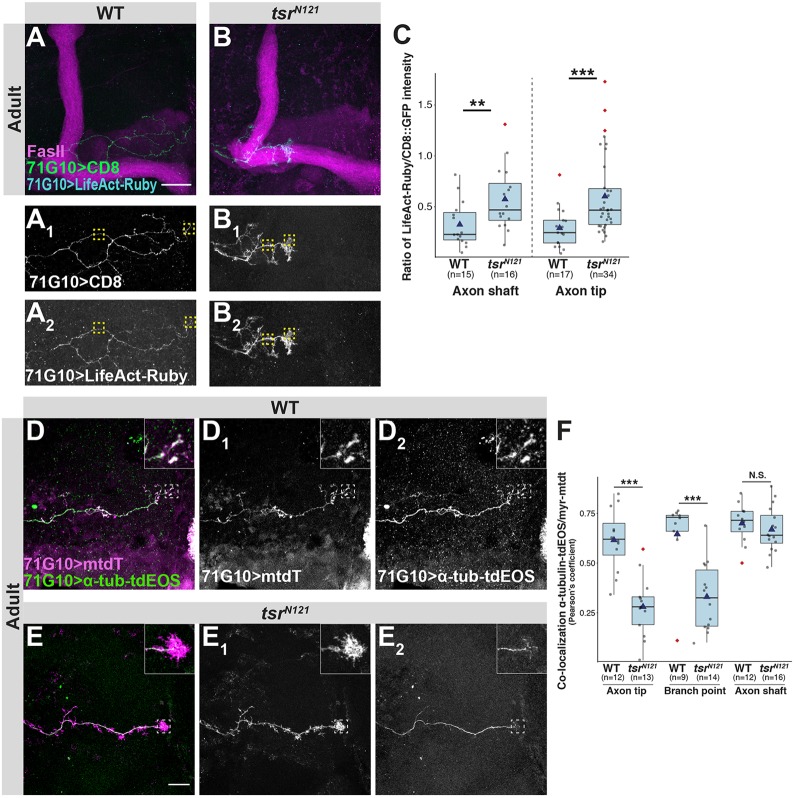
**Microtubule localization is perturbed in *tsr* mutants.** (A,B) Confocal *Z*-projections of WT (A) or *tsr^N121^* (B) adult γ-neuron MARCM SCCs. Yellow dashed squares demark representative regions used for quantification of LifeAct intensity in the axon shaft and tip. Green and cyan are R71G10-Gal4-driven mCD8::GFP or LifeAct-Ruby, respectively. Gray is R71G10-Gal4-driven mCD8::GFP (A_1_,B_1_) or LifeAct-Ruby (A_2_,B_2_). Magenta is FasII staining. Scale bar: 20 µm. (C) Box-plot quantification of LifeAct to CD8::GFP intensity ratio in marked regions in A,B. (D,E) Confocal *Z*-projections of WT (D) or *tsr^N121^* (E) adult γ-neuron MARCM SCCs. Insets correspond to dashed boxes as examples for regions used for colocalization analyses – in this case, an axon tip. Magenta and green are R71G10-Gal4-driven mtdT or α-tubulin84B-tdEOS, respectively. Gray is R71G10-Gal4-driven mtdT (D_1_,E_1_) or α-tubulin84B-tdEOS (D_2_,E_2_). Scale bar: 10 µm. (F) Box-plot quantification of mtdT (membranes) and α-tubulin84B-tdEOS (MT) colocalization. In each neuron at least one axon tip, branch point and axon shaft were quantified. Colocalization was tested using Pearson's coefficient. While in *tsr^N121^* there is less colocalization in both the axon tip and branch point, there is no difference in the main axon shaft. **P<0.01; ***P<0.005; N.S., not significant.

We next turned to examine whether Tsr is required for the elimination of actin structures, thereby allowing MT elongation (Flynn et al., 2012; Tedeschi et al., 2019). First, we determined that F-actin levels were indeed increased within *tsr* SCCs compared to WT (Fig. 4A–C). Next, we co-expressed membrane-bound tandem tomato (mtdT) and a tubulin reporter (α-tubulin84B.tdEOS), which, in WT SCCs, demonstrated that MTs and membrane markers seem to overlap (Fig. 4D). In contrast, MTs did not seem to extend into the enlarged filopodia-like structures (Fig. 4E) in *tsr* mutants. Interestingly, quantification of the colocalization (assessed by the Pearson's coefficient) revealed that colocalization was decreased in both axon branch points and tips but remained unaffected in the main axon shaft (Fig. 4F). These results suggest that Tsr severs F-actin to clear actin aggregates, which, in turn, is crucial for proper MT protrusion. To demonstrate this in a more direct manner, we attempted to express the MT reporter α-tubulin84B.tdEOS in combination with the F-actin reporter F-tractin but unfortunately due to rapid bleaching were unable to extract meaningful high-resolution images. Therefore, while our findings support the hypothesis that Tsr reduces F-actin accumulations to allow MT elongation, further exploration is necessary. These findings are consistent with *in vitro* studies demonstrating increased F-actin levels in ADF/cofilin mutants. These increased F-actin levels resulted in accumulations that were suggested to obstruct and misdirect MTs causing a decrease in neurite formation (Flynn et al., 2012).

To conclude, we have delved deeper into the role of Tsr during axon formation, including in regrowth and branching. We have followed up on several *in vitro* studies, and showed that Tsr regulates growth and branching *in vivo*, presumably by severing F-actin thereby allowing the protrusion of MTs into the potential growth cone.

## MATERIALS AND METHODS

### Generation of MARCM clones

MARCM clones of MB neurons and single cell clones were generated by a 1 h heat-shock of newly hatched larvae (24 h after egg laying) and examined at the indicated developmental stages, as described previously (Lee et al., 1999).

### Immunostaining and imaging

*Drosophila* brains were dissected in cold Ringer's solution, fixed using 4% paraformaldehyde for 20 min at room temperature (RT), and washed in phosphate buffer with 0.3% Triton-X (PBT) (three immediate washes and three 20-min washes). Non-specific staining was blocked by 5% inactivated goat serum in PBT. Brains were subjected to primary antibody overnight at 4°C. Primary antibodies included: chicken anti-GFP (GFP-1020; AVES), 1:500; and mouse monoclonal anti-FasII (1D4), 1:25 (Developmental Studies Hybridoma Bank; DSHB). Brains were washed with PBT (three immediate washes and three 20-min washes), and then stained with secondary antibodies for 2 h at room temperature. Secondary antibodies included: goat Alexa Fluor 647-conjugated anti-mouse-IgG (A-21236; Invitrogen), 1:300; and donkey FITC-conjugated anti-chicken-IgG (703-095-155; Jackson Immunoresearch), 1:300. Brains were mounted on Slowfade (S-36936; Invitrogen) and imaged on Zeiss LSM 800 confocal microscope. Images were processed with ImageJ (NIH).

### *Drosophila melanogaster* rearing and strains

All fly strains were reared under standard laboratory conditions at 25°C on molasses-containing food. Males and females were chosen at random. Unless specifically stated otherwise, the relevant developmental stage is adult, which refers to 3–5 days post eclosion.

*tsr^N121^*, *tsr^N96A^*, UAS-tsr.N, UAS-tsr.S3A, UAS-tsr.S3E, UAS-act5c, UAS-LifeAct-Ruby, UAS-F-tractin.tdTomato, UAS-α-tubulin84B-tdEOS and RNAi lines (*cpa, cpb, chic*) were all obtained from the Bloomington *Drosophila* stock center (Indiana University, USA). UAS-Chic was kindly provided by Florence Besse (Institut de Biologie Valrose, Nice, France) (Medioni et al., 2014).

*Drosophila* genotypes used in this study are listed in Table S1.

### Sprouting assay

Dissociation and plating of cells has been previously described (Marmor-Kollet and Schuldiner, 2016). Briefly, L3 brains were dissociated in 10 mg/ml collagenase (Sigma), washed three times with PBS and resuspended in Schneider's medium (Sigma) supplemented with 10% heat-inactivated fetal bovine serum and antibiotics. Dissociated cells were plated in glass-bottom 96-well plates coated with poly-L-lysine (MatTek) at ∼10 brains/well in a volume of ∼30 µl in culturing medium. At 1 h after plating, medium was added to give a total volume of 200 µl. Fluorescent cells were imaged 2 days after plating (DIV) and neurite length was calculated using the Simple Neurite Tracer plugin in Fiji. When indicated, 10 µM latrunculin B or 10 µM jasplakinolide (Life Technologies) were added 1 DIV and incubated an additional 24 h before imaging.

Sprouting live imaging was performed on cultured neurons labeled with CD8:GFP and Ftractin-tdT starting at 48 h *in vitro* (2DIV). They were imaged over a period of 10 h, at 15 min intervals. For analysis of dynamics, total neurite length was measured at every 15 min interval (using the Simple Neurite Tracer plugin) for a period of 3 h. Changes between time points were summed for each 1 h period and averaged over 3 h.

### Statistical analysis

We strove for *n*>10 brain hemispheres; *n* values are shown in the quantifications within the figures. In the sprouting experiments, *n* represents cells. No samples were excluded from the analyses.

For the quantification of developmental regrowth in MARCM clones (Fig. 1M), we used a method previously described (Yaniv et al., 2012). In short, we determined the γ lobe occupancy by comparing the clonal (GFP) versus non clonal (FasII staining) in the Z-plane cross section. To calculate the regrowth index, we then divided the lobe occupancy of the clonal axons at a distal section by a proximal section.

For analysis of SCCs (Figs 1N and 3H), the furthest point of innervation into adult γ lobe was measured on the confocal *Z*-stack and divided by the total length of the γ lobe (determined by FasII staining). See Fig. S1F for details.

For *in vivo* branching (Fig. 2D), the number of secondary branches over >5 µm were counted and normalized to 100 µm of axon length.

For LifeAct–Ruby intensity studies (Fig. 4C), in each image the intensity of both LifeAct–Ruby and GFP were measured at two different points along the axon as well as at the axon tip and normalized to the background.

For MT and membrane-bound RFP colocalization studies (Fig. 4F), in each neuron, at least two branch points, two axon terminals (if available) and two points along the main axon were measured for both GFP and RFP. Colocalization, represented by Pearson's coefficient, was measured using the Coloc2 Fiji plugin.

Statistical analysis was performed by a one-way ANOVA including all groups followed by a Tukey post-hoc test (Figs 1M,N and 3H) or Student's *t*-test (Figs 2G–I and 4C,F).

For all box plots, boxes encompass the values in between the 1st–3rd quartiles; whiskers are ±1.5 interquartile range (IQR), the median is represented by the line, the mean by the blue triangle, and outliers by red diamonds.

## Supplementary Material

Supplementary information
