# Supplementary material for: Cofilin regulates axon growth and branching of Drosophila γ-neurons
Source: J Cell Sci. 2020 Apr 28;133(8):jcs232595. doi: 10.1242/jcs.232595 (PMC7197873; doi:10.1242/jcs.232595)
Supplement: Supplementary information [file joces-133-232595-s1.pdf]

Figure S1

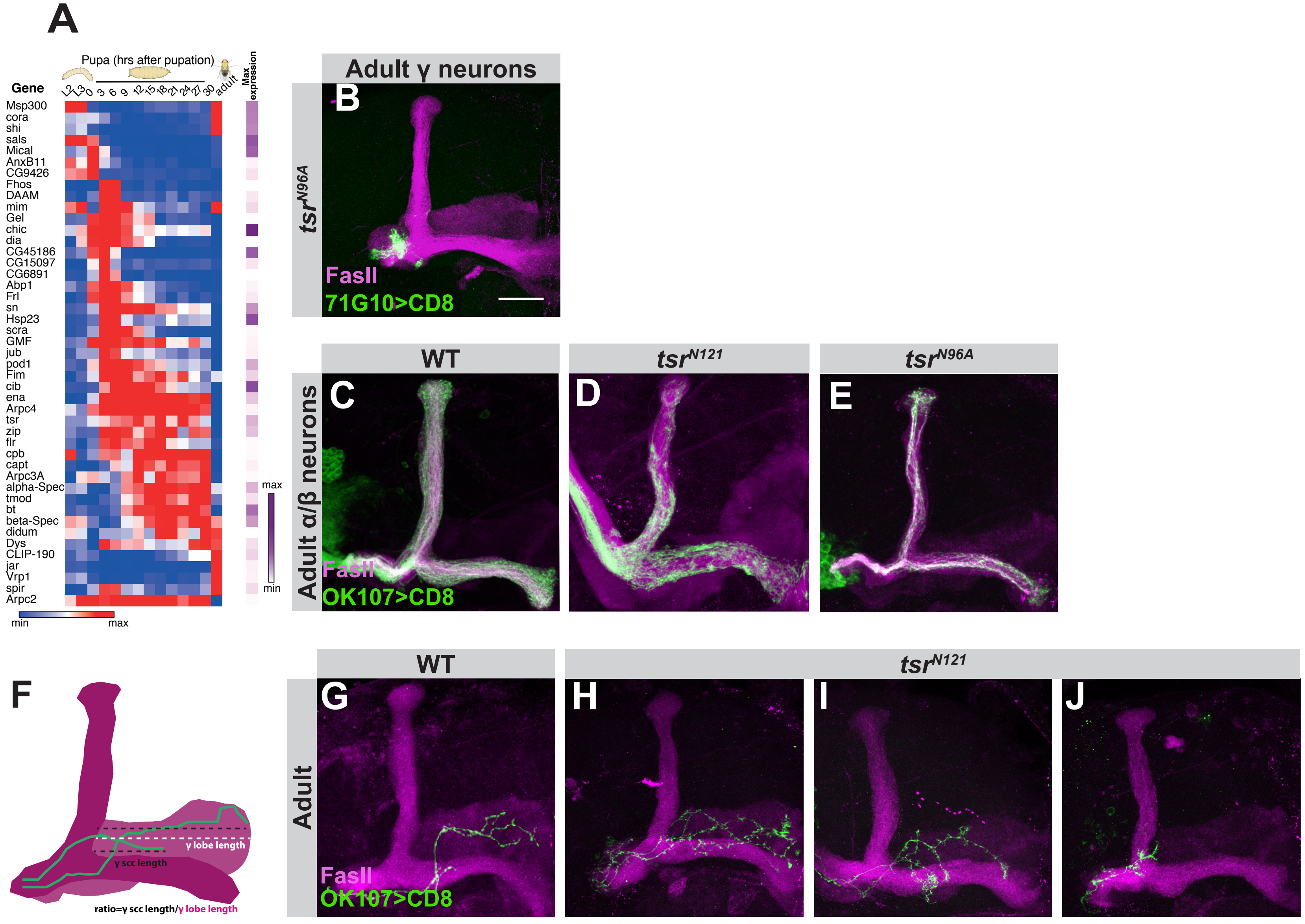

Journal of Cell Science • Supplementary information

Figure S1: Expression pattern of actin related genes, phenotype of *tsr*^{N96A} and phenotypes of tsr in α/β neurons

(A) Heatmap depicting the relative RNA expression levels of genes relevant to actin dynamics throughout development from 2nd instar larva (L2) to adult. The purple scale depicts the peak expression of each gene relative to others presented in the scheme. Genes are ordered based on their clustered expression - see Alyagor et al (2018) for technical details.

(B) Confocal z-projections of adult *tsr*^{N96A} MB γ neuron MARCM neuroblast clones.

(C-E) Confocal Z projections of WT (C), *tsr*^{N121} (D) or *tsr*^{N96A} (E) in adult MARCM neuroblast clones of α/β neurons.

(F) Scheme depicting the quantification of single cell clone phenotype severity: In each Z projection, the relative length of the labelled single cell out of the entire lobe was calculated.

(G-J) Confocal z-projections of adult MB γ neuron MARCM single cell clones in WT (G) or *tsr*^{N121} displaying ohenotypic variability (H-J).

Green is R71G10-Gal4 (B) or OK107-Gal4 (C-D, G-J) driven mCD8::GFP. Magenta is FasII staining. Scale bar is 20µm.

Journal of Cell Science • Supplementary information

Figure S2

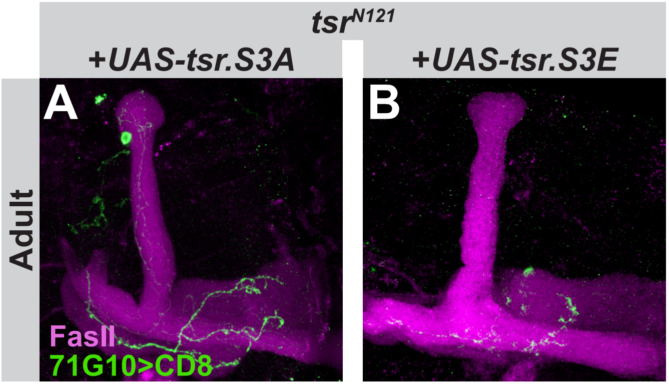

Figure S2: Axon growth rescue experiments demonstrate the importance of phosphoregulation of Tsr

(A-B) Confocal Z projections of γ neuron MARCM single cell clones of *tsr*^{N121} additionally expressing *UAS-tsr.S3A* (A) or *UAS-tsr.S3A* (B). Quantification shown in in Fig 1N. Green is R71G10-Gal4 driven mCD8::GFP. Magenta is FasII staining

Journal of Cell Science • Supplementary information

Figure S3

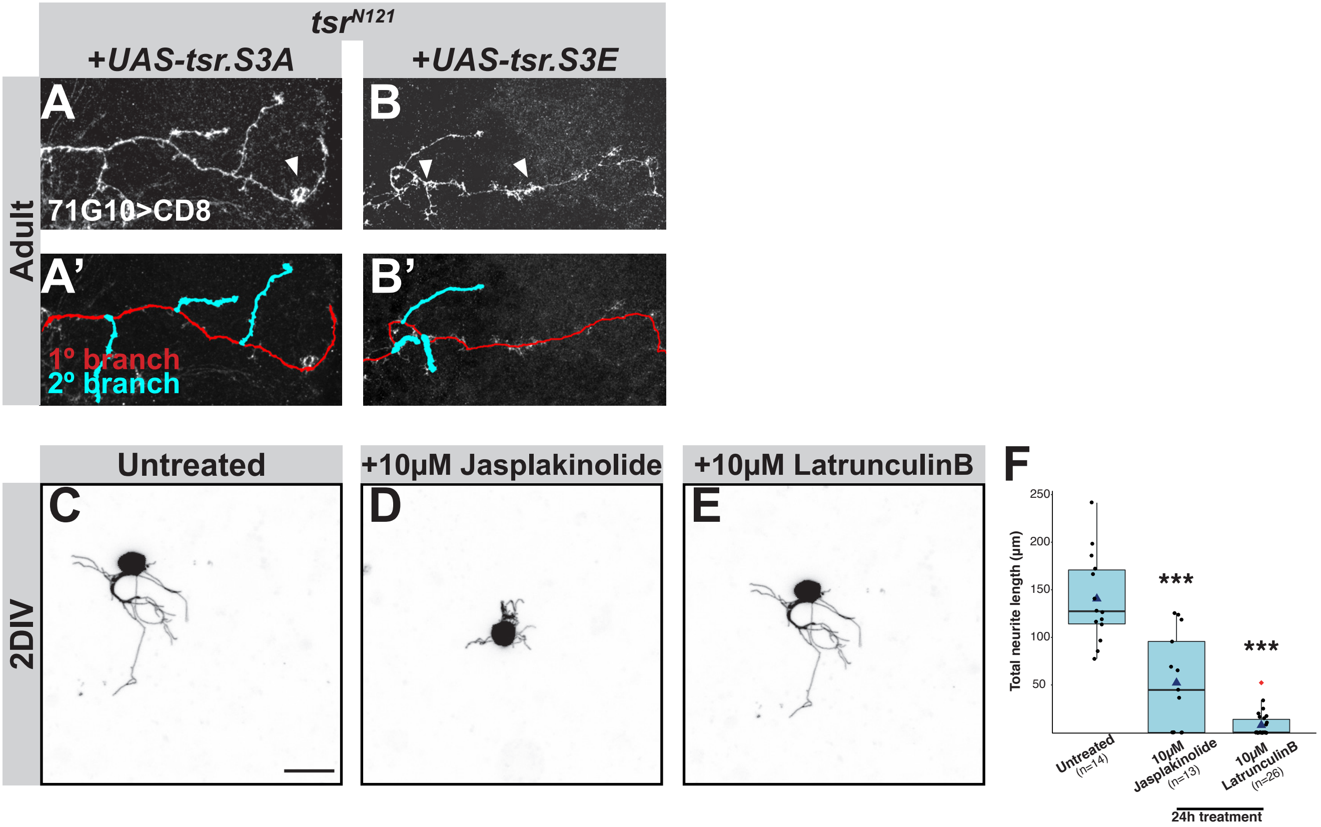

<u>Figure S3: Branching rescue experiments *in vivo*, and pharmacological manipulations of sprouting neurons</u>

(A-B) Confocal Z projections of γ neuron MARCM single cell clones of *tsr*^{N121} additionally expressing *UAS-tsr.S3A* (A) or *UAS-tsr.S3A* (B). Top panels focus on the γ axon region, bottom panel is same image with the primary axon labeled in red and secondary branches labeled in cyan. Arrowheads point to structures within the axon that appear to be failed branch points. Grey is R71G10-Gal4 driven mCD8::GFP. Scale bar is 20µm

(C-E) Confocal Z projections or primary γ neurons derived from L3 brains untreated (C), or treated with 10µM Jasplakinodine for 24h (D) or 10µM LatrunculinB for 24h (E).

(F) Box plot quantifications of C-E: total neurite length per cell.

Figure S4

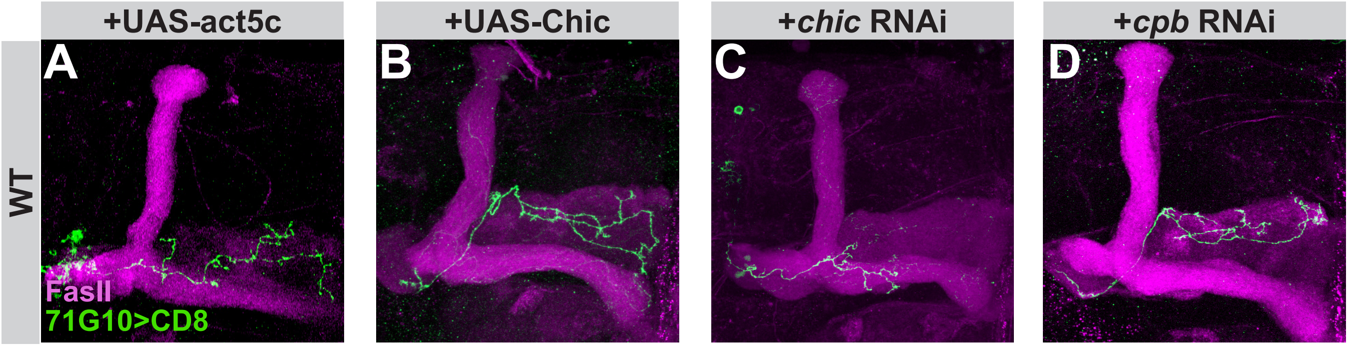

Figure S4: Control experiments of actin and capping protein perturbations in Tsr WT animals

(A-D) Confocal Z projections of MB γ neurons expressing UAS-act5c (A), UAS-Chic (B), *chic* RNAi (C), *cpb* RNAi (D). Green is R71G10-Gal4 driven mCD8::GFP. Magenta represents FasII staining. Scale bar is 20µm

Journal of Cell Science • Supplementary information

Table S1. Drosophila Genotypes used in this study

hsFLP is y,w,hsFLP122; CD8 is UAS-mCD8::GFP; mtdT is UAS-myristoylated tdTomato; G13 is FRT on 2R; Gal80 is TubP-Gal80. Males and females were used interchangeably but only the female genotype is mentioned.

Figure	Panels	Detailed genotype
**Figure 1**	B, E, H, J	hsFlp, CD8/+; 71G10-Gal4, G13, Gal80/G13
	C, F, I, K	hsFlp, CD8/+; 71G10-Gal4, G13, Gal80/G13, *tsr*^{N121}
	D, G, L	hsFlp, CD8/+; 71G10-Gal4, G13, Gal80/G13, *tsr*^{N121}; UAS-tsr.N/+
**Figure 2**	A	hsFlp, CD8/+; 71G10-Gal4, G13, Gal80/G13
	B	hsFlp, CD8/+; 71G10-Gal4, G13, Gal80/G13, *tsr*^{N121}
	C	hsFlp, CD8/+; 71G10-Gal4, G13, Gal80/G13, *tsr*^{N121}; UAS-tsr.N/+
**Figure 3**	A	hsFlp, CD8/+; 71G10-Gal4, G13, Gal80/G13
	B	hsFlp, CD8/+; 71G10-Gal4, G13, Gal80/G13, *tsr*^{N121}
	C	hsFlp, CD8/+; 71G10-Gal4, G13, Gal80/G13, *tsr*^{N121}; UAS-act5c/+
	D	hsFlp, CD8/+; 71G10-Gal4, G13, Gal80/G13, *tsr*^{N121}; UAS-chic/+
	E	hsFlp, CD8/+; 71G10-Gal4, G13, Gal80/G13, *tsr*^{N121}; UAS-chic RNAi/+
	F	hsFlp, CD8/+; 71G10-Gal4, G13, Gal80/G13, *tsr*^{N121}; UAS-cpa RNAi/+
	G	hsFlp, CD8/+; 71G10-Gal4, G13, Gal80/G13, *tsr*^{N121}; UAS-cpb RNAi/+
**Figure 4**	A	hsFlp, CD8/+; 71G10-Gal4, G13, Gal80/G13; UAS-LifeAct-Ruby/+
	B	hsFlp, CD8/+; 71G10-Gal4, G13, Gal80/G13, *tsr*^{N121}; UAS-LifeAct-Ruby/+
	D	hsFlp, mtdT/+; 71G10-Gal4, G13, Gal80/G13; UAS-α-tubulin84B-tdEOS/+
	E	hsFlp, mtdT/+; 71G10-Gal4, G13, Gal80/G13, *tsr*^{N121}; UAS-α-tubulin84B-tdEOS/+
**Figure S1**	A	hsFlp, CD8/+; 71G10-Gal4, G13, Gal80/G13, *tsr*^{N96A}
	C	hsFlp, CD8/+; G13, Gal80/G13; OK107-Gal4/+
	D	hsFlp, CD8/+; G13, Gal80/G13, *tsr*^{N121}; OK107-Gal4/+
	E	hsFlp, CD8/+; G13, Gal80/G13, *tsr*^{N96A}; OK107-Gal4/+
	G	hsFlp, CD8/+; 71G10-Gal4, G13, Gal80/G13
	H-J	hsFlp, CD8/+; 71G10-Gal4, G13, Gal80/G13, *tsr*^{N121}
**Figure S2**	A	hsFlp, CD8/+; 71G10-Gal4, G13, Gal80/G13, *tsr*^{N121}; UAS-tsr.S3A/+
	B	hsFlp, CD8/+; 71G10-Gal4, G13, Gal80/G13, *tsr*^{N121}; UAS-tsr.S3E/+

Journal of Cell Science • Supplementary information

**Figure S3**	A	hsFlp, CD8/+; 71G10-Gal4, G13, Gal80/G13, *tsr*^{N121}; UAS-tsr.S3A/+
	B	hsFlp, CD8/+; 71G10-Gal4, G13, Gal80/G13, *tsr*^{N121}; UAS-tsr.S3E/+
**Figure S4**	A	71G10-Gal4, CD8/+; *cpb* RNAi/+
	B	71G10-Gal4, CD8/+; UAS-act5c/+
	C	71G10-Gal4, CD8/+; UAS-Chic/+
	D	71G10-Gal4, CD8/+; *chic* RNAi/+

Journal of Cell Science • Supplementary information

Supplemental movies

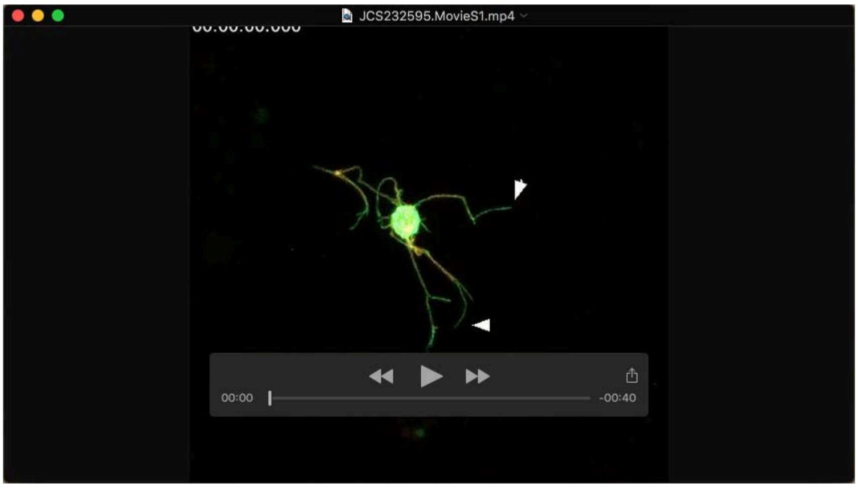

Movie 1:

Confocal Z projections of live imaging of WT primary cells derived from L3 brains starting at 2 days in vitro (2DIV). Cells were imaged over a period of 10 hours, at 15-minute intervals. White arrows mark extending or retracting neurite.

Green is R71G10-Gal4 driven mCD8:GFP. Red is R71G10-Gal4 driven F-tractin.tdTomato.

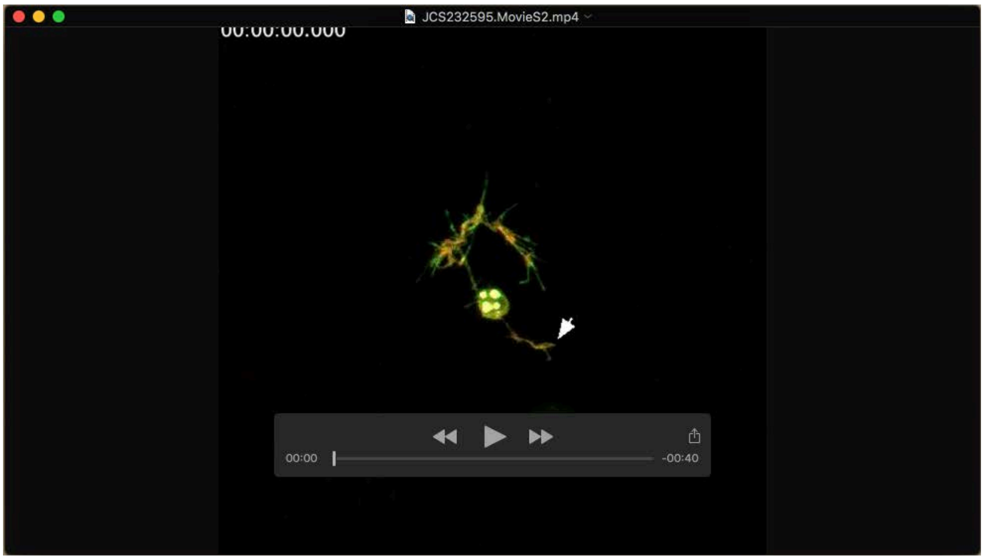

Movie 2:

Confocal Z projections of live imaging of *tsr*^{N121} primary cells derived from L3 brains starting at 2DIV. Cells were imaged over a period of 10 hours, at 15-minute intervals. White arrows mark extending or retracting neurite.

Green is R71G10-Gal4 driven mCD8:GFP. Red is R71G10-Gal4 driven F-tractin.tdTomato.

Journal of Cell Science • Supplementary information
